# The application of traffic psychology scales in assessment for fitness-to-drive of individuals with mental disorders

**DOI:** 10.1192/j.eurpsy.2025.606

**Published:** 2025-08-26

**Authors:** S. Wang, X. Ling, Q. Zhang, H. Li

**Affiliations:** 1 Shanghai Key Lab of Forensic Medicine, Key Lab of Forensic Science, Ministry of Justice, Shanghai Forensic Service Platform, Academy of Forensic Science, Shanghai, China

## Abstract

**Introduction:**

Mental disorder may affect individual’s ability to operate the motor vehicle. Previous studies have found that patient’s negative emotions may trigger aggressive driving behaviors. Thus, efficiently evaluating the correlation between emotions and driving behaviors in individuals with mental disorders has been drawn emphasis.

**Objectives:**

To explore the related factors of fitness-to-drive of individuals with mental disorders, to determine the application value of traffic psychology scales in assessment for fitness-to-drive of individuals with mental disorders, and to help establish consummate and effective assessment systems.

**Methods:**

One hundred individuals with mental disorders were enrolled as the patient group, and 100 healthy individuals were enrolled as the control group. Positive and Negative Syndrome Scale (PANSS) was used to assess the psychiatric symptoms of the patient group. Driver Profile of Mood States (DPOMS), Driver Anger Scale (DAS), and Driving Behavior Scale (DBS) were used to evaluate the performance during driving within two groups. T-test were used to compare the differences in each factor score of traffic psychology scales within two groups. Pearson’s correlation analysis was used to calculate the correlation between scores of PANSS and scores of traffic psychology scales of the patient group.

**Results:**

The patient group had significantly higher score of driving function deficit in DBS than the control group (t=2.48, P<0.05), but scores of hostile gestures, impolite driving, overly cautious behaviors in DBS and total score of DAS showed the opposite (P<0.05). Positive syndrome in PANSS was positively related to traffic congestion in DAS (r = 0.315, P < 0.05). Anger in DPOMS was positively related to driving function deficit (r = 0.488, P < 0.01) and hostile behaviors in DBS (r = 0.510, P < 0.01), whereas it was negatively related to overly cautious behaviors in DBS (r = -0.417, P < 0.05). Anxiety and depression were also related to some factors in DAS and DBS.

**Conclusions:**

The study found the practical application value of DPOMS, DAS, and DBS in assessment for fitness-to-drive of individuals with mental disorders. Patient’s anger in specific traffic situations such as traffic congestion may be mainly related to their positive syndrome. Patient’s anger may be a trigger of aggressive driving behaviors, and other emotions such as anxiety and depression also play important roles. Patient’s aggressive driving behaviors may be attributed to the compounding of many negative emotions.

**Disclosure of Interest:**

S. Wang: None Declared, X. Ling: None Declared, Q. Zhang: None Declared, H. Li Grant / Research support from: This study was supported by National Key R & D Program of China [grant number 2022YFC3302001], National Natural Science Foundation of China [grant number 81801881], Science and Technology Committee of Shanghai Municipality [grant numbers 20DZ1200300, 21DZ2270800, 19DZ2292700].

